# Harmonized food consumption dataset by food category and acquisition source for Sub-Saharan African countries

**DOI:** 10.1038/s41597-026-06548-1

**Published:** 2026-01-21

**Authors:** Amaka P. Nnaji, Didier Yelognisse Alia, Ahana Raina, C. Leigh Anderson

**Affiliations:** Evans School Policy Analysis Research (EPAR) group, Daniel J. Evans School of Public Policy and Governance, University of Washington, Seattle, WA USA

**Keywords:** Agriculture, Economics

## Abstract

Household consumption is a key measure of well-being in low- and middle-income countries (LMICs) where agriculture remains the primary livelihood, and food represents a substantial share of household total consumption expenditures. This paper introduces a new harmonized dataset of food consumption value by food categories and acquisition sources for 16 sub-Saharan African countries from 2008 to 2021. The dataset is constructed from consumption modules of large-scale, nationally representative household surveys collected by the World Bank and each country’s National Statistical Office. It adds value to these surveys by standardizing indicators, including monetizing non-market consumption, generating food item-level estimates, and making the processing code and record-level microdata publicly available for replication and use by researchers. The dataset facilitates valid cross-country comparisons of food consumption over time and can be merged with other satellite and climate data datasets for additional analysis of the drivers and impacts of food consumption in LMICs. Additionally, an indicator dashboard and visualizations have been created to make the estimates accessible to policymakers and the public.

## Background & Summary

In most low- and middle-income countries (LMICs), household consumption is an important indicator of household wellbeing^1–3^. For several decades, prior to the COVID-19 pandemic, household consumption in many of these countries increased substantially and global poverty fell^4–6^. Increases in household consumption have been driven by a host of factors that include rising incomes, globalization, and increased economic integration^7–9^. In most LMICs, food consumption constitutes the largest share of household total consumption. In sub-Saharan Africa (SSA), household food preferences are also changing due to increased urbanization, education levels, and rising income. Studies have documented a shift away from the consumption of staple foods toward an increasingly diversified diet^10,11^. These changes have implications for household nutrition and agricultural transformation. Hence, it is important to understand the dynamics of household food consumption across different food items and food sources.

Most studies on household food consumption patterns are limited to a single country^7,12–15^. One of the constraints to cross-country analysis of food consumption patterns is the lack of comparable datasets. While most countries conduct national household living standard surveys, there are barriers to using these data. For example, most publicly available surveys with consumption modules are inconsistent between countries. Also, formulations of food consumption questions in these surveys are inconsistent across countries and sometimes vary across different survey rounds of the same country. For example, food consumption data collected in the following surveys: the Madagascar Enquête Périodique auprès des Ménages (EPM), the Rwanda Integrated Household Living Conditions Survey (EICV), and the South Africa Income and Expenditure Survey were not disaggregated by source of acquisition. Additionally, some existing datasets only report aggregate food consumption, which leaves little room for micro-level disaggregated information. For instance, the 2011 Senegal Enquête de Suivi de la Pauvreté (ESPS-II) survey did not collect disaggregated crop-level food consumption data. Standardizing food consumption indicators across countries and multiple waves of publicly available data would facilitate analyses of trends in the value of different food categories consumed and their sources.

This paper introduces a newly harmonized dataset of food consumption indicators by food category and acquisition source for 16 SSA countries over the period 2008–2021. The dataset was constructed using publicly available nationally representative household surveys collected by the World Bank’s Living Standards Measurement Study - Integrated Surveys on Agriculture (LSMS-ISA) team and individual country’s National Statistical Offices. Some countries have collected data over multiple rounds, including panel data, following the same household over multiple years. Information can be further aggregated at the household, regional, and country levels. The dataset also includes key household demographic characteristics like gender and age of household head, location of household, household size, and dependents. This dataset contributes to and complements recent efforts to make comparable cross-country socio-economic and agricultural production data for LMICs public and more widely available^16–21^. These data were originally prepared by the Evans School Policy and Analysis group (EPAR) to provide nutritional insights from changes in food groups consumed, but will be useful for researchers, analysts, and policymakers seeking to understand food consumption trends across multiple countries and years. This paper outlines the data processing steps in constructing the harmonized food consumption dataset, a description of the data structure, and usage recommendations.

## Methods

### Data sources and sample

We focus on processing and standardizing food consumption data from publicly available surveys, ensuring full compliance with their terms of use. Table 1 presents the list of survey data processed. It includes the name of the survey and the years of data collection. The processing covers 36 surveys collected from 16 SSA countries between 2008 and 2021. Table S1 in the Supplementary Information provides inclusion and exclusion criteria for countries in this harmonized dataset, and Table S2 provides links to the raw data for each included survey.

**Table 1:** Source Datasets, Countries, and Years Covered.

Country	Survey	Waves
Benin	Enquête Harmonisée sur les Conditions de Vie des Ménages (EHCVM)	2018/19^26^
Burkina Faso	Enquête Harmonisée sur les Conditions de Vie des Ménages (EHCVM)^a^	2018/19^27^, 2021/22^28^
Cote d’Ivoire	Enquête Harmonisée sur les Conditions de Vie des Ménages (EHCVM)	2018/19^29^
Ethiopia	Socioeconomic Panel Survey (ESS)^b^	2015/16^30^, 2018/19^31^, 2021/22^32^
Ghana	Socioeconomic Panel Survey (SPS)	2009/10^33^
Guinea Bissau	Enquête Harmonisée sur les Conditions de Vie des Ménages (EHCVM)	2018/19^34^
Kenya	Integrated Household Budget Survey (KIHBS)	2015/16^35^
Malawi	Integrated Household Survey (IHS)^c^	2010/11^36^, 2013^37^, 2016/17^38^, 2019/2020^39^
Mali	Enquête Agricole de Conjoncture Intégrée (EACI)	2014^40^
	Enquête Harmonisée sur les Conditions de Vie des Ménages (EHCVM)	2018/19^41^
Niger	Enquête Harmonisée sur les Conditions de Vie des Ménages (EHCVM)	2018/19^42^
Nigeria	General Household Survey (GHS)^d^	2010/12^43^, 2012/13^44^, 2015/16^45^, and 2018/19^46^
Senegal	Enquête Harmonisée sur les Conditions de Vie des Ménages (EHCVM)	2018/19^47^
Sierra Leone	Integrated Household Survey (IHS)	2018^48^
Tanzania	National Panel Survey (NPS)^e^	2008/09^49^, 2010/11^50^, 2012/13^51^, 2014/15^52^, 2019/20^53^
Togo	Enquête Harmonisée sur les Conditions de Vie des Ménages (EHCVM)	2018/19^54^
Uganda	National Panel Survey (UNPS)^f^	2009/10^55^, 2010/11^56^, 2011/12^57^, 2013/14^58^, 2015/16^59^, 2018/19^60^, 2019/20^61^

Notes: (a) The two Burkina Faso EHCVM waves are panel datasets; (b) Ethiopia ESS 2018/19 and 2021/22 are panel datasets; (c) The four Malawi IHS waves are panel datasets; (d) The Nigeria GHS 2010/12, 2012/13, and 2015/16 waves are panel datasets, while the 2018/19 wave is a refreshed sample that only tracked a subset of the original panel sample; (e) The Tanzania NPS 2008/09, 2010/11, 2012/13, and 2014/15 waves are panel datasets, the 2019/20 wave is a refreshed sample that only tracks a subset of the original panel sample; (f) The Uganda 2009/10, 2010/11, and 2013/14 waves can be merged into a panel, while the other waves are refreshed and track only a subset of the original sample. All the other surveys are cross-sectional or repeated cross-sectional datasets

**Fig. 1:**
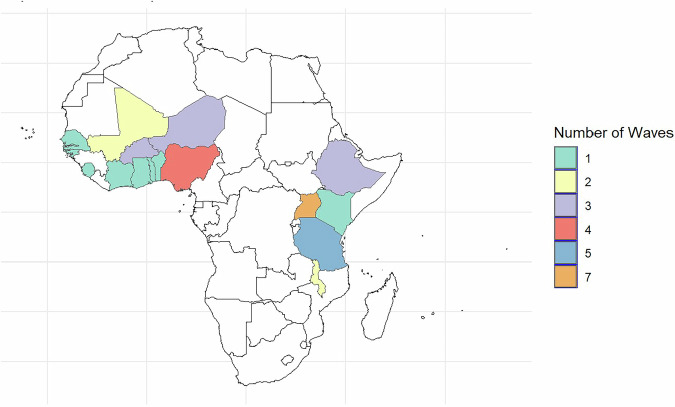
Countries covered and the number of rounds of data availability.

**Fig. 2:**
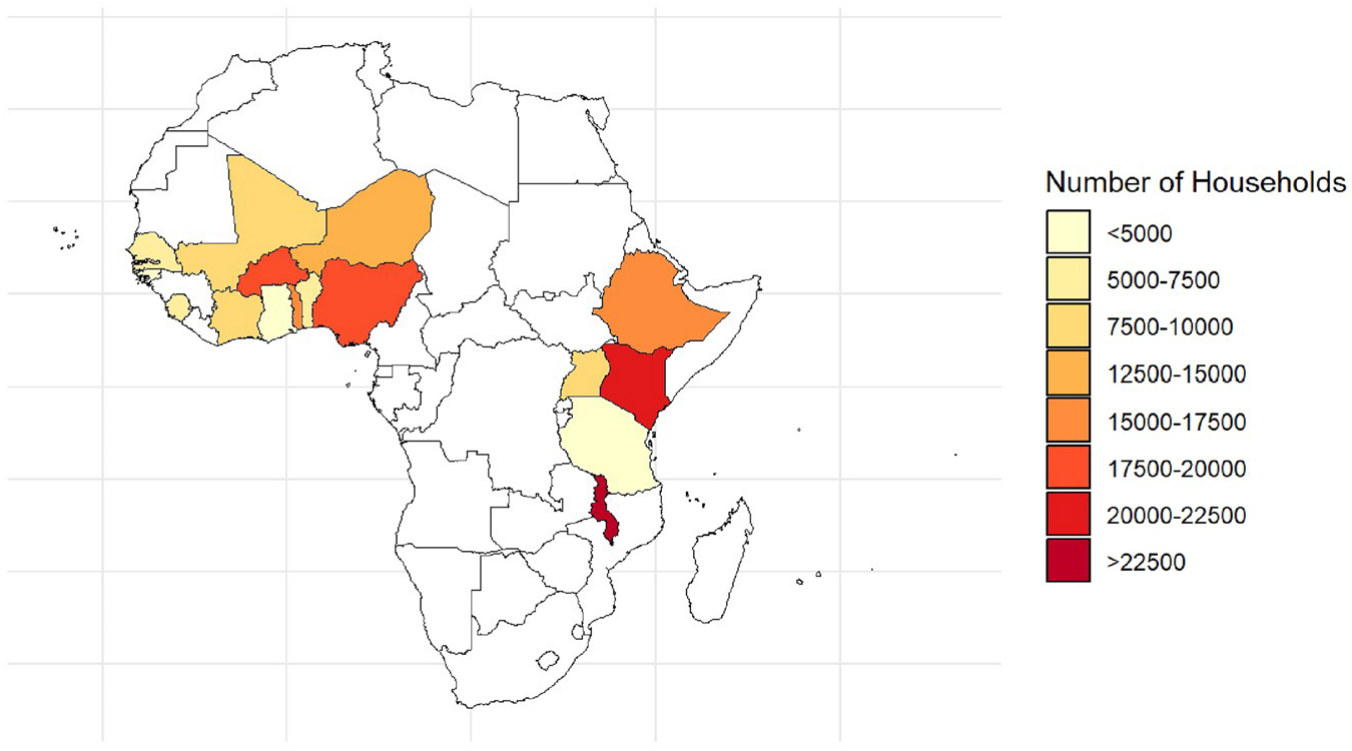
Number of households captured by country across all surveys.

The 16 countries included in the dataset had a combined Gross Domestic Product (GDP) of about US$1057 billion in 2021, representing about 54% of the total GDP of SSA for that year^22^. They also accounted for about 703 million individuals or 59% of the total SSA population. Thus, the data provides a broad representation of the continent, both in terms of GDP and population. Figure 1 below shows a map of the countries included in the dataset, with color shades representing the number of rounds for which data are available. Figure 2 presents the same map but color-codes the number of households captured in each country, ranging from 5003 in Ghana to over 30,000 households in Malawi. Figure 2 reflects the total number of household-level data points across all waves in each country, not necessarily unique as, households may appear multiple times in countries with panel data.

#### Data processing

**Fig. 3:**
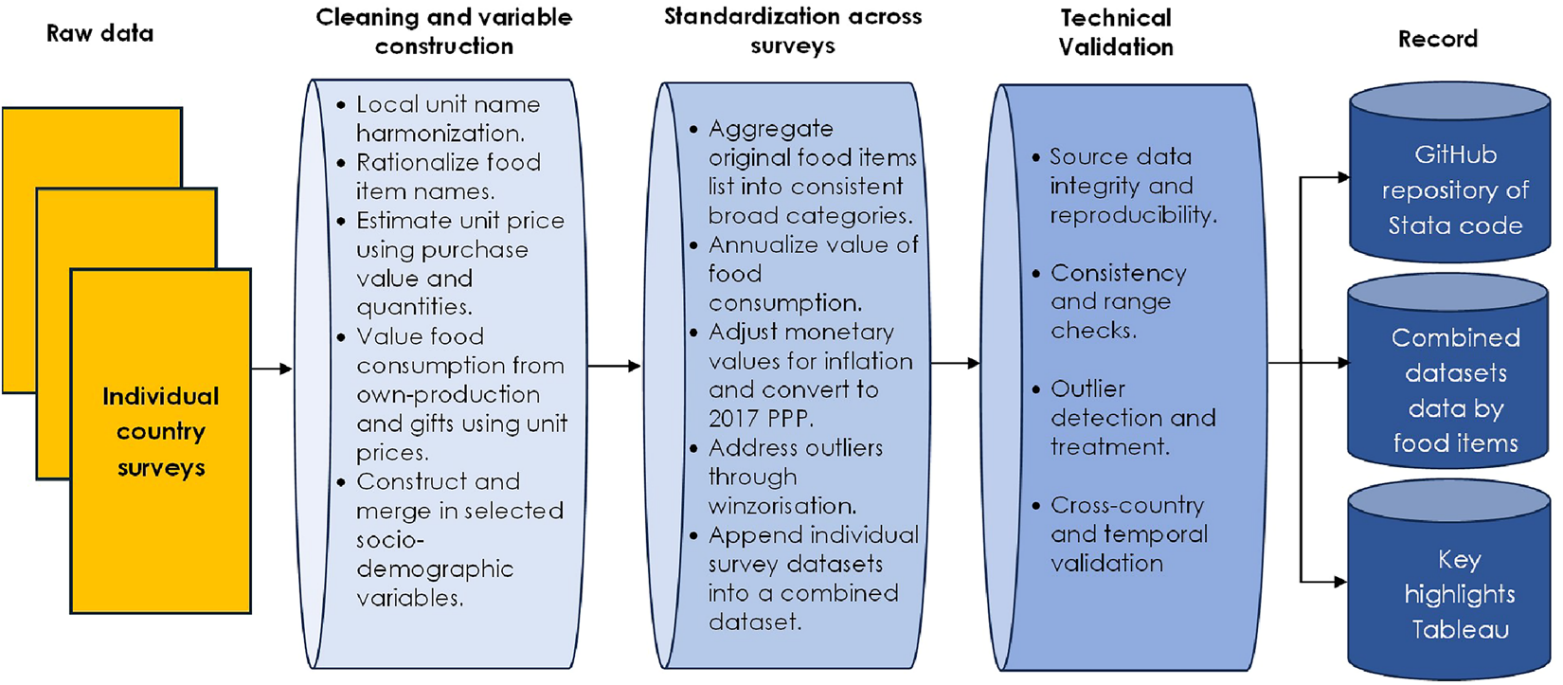
Data processing workflow.

We add value to publicly available consumption data by constructing comparable indicators across countries and years. The consumption modules in these surveys are not standardized; hence, processing the data involves several steps. Figure 3 summarizes these steps.

We begin by downloading the raw data files from the websites of the World Bank or the country’s national statistical agencies (Table S2). The raw data consists of questions, enumerated across different years, asking households about the quantity of food consumption from their own production and gifts, as well as the amount and value of food purchased over a period of time. In each survey, we combine several questions from the consumption module to construct estimates of food consumption value by food items and sources of acquisition. Survey questions vary across countries, sometimes even for the same country across waves. Recall period, the food items included in the basket of goods, and how items are enumerated and aggregated can all vary. For example, questions in the Nigeria LSMS-ISA/General Household Survey consumption Wave 3 module use a 7-day recall and ask about quantity and unit by source, and expenditure on purchases only:

- How much in total did your household consume of this [ITEM] (quantity)
- How much in total did your household consume of this [ITEM] (unit)
- How much did your household purchase of [ITEM]? (quantity)
- How much did your household purchase of [ITEM]? (unit)
- How much did your household spend on [ITEM](NAIRA)
- How much of the consumption of [ITEM] comes from purchases? (quantity)
- How much of the consumption of [ITEM] comes from purchases? (unit)
- How much consumption of [ITEM] came from own production? (quantity)
- How much consumption of [ITEM] came from own production? (unit)
- How much consumption of [ITEM]came from gift? (quantity)
- How much consumption of [ITEM]came from gift? (unit)

In contrast, in the Ghana Socioeconomic Panel Survey (SPS), the recall period is 30 days, the quantity consumed from different sources is reported in the same unit, and respondents were asked to self-estimate and report the monetary value of consumption from own production, purchase, and gifts in Ghana Cedis. Although the Ghana SPS raw data includes households’ self-estimated value of food consumption from own production, and gifts, for consistency, we reconstructed these estimates following the same data processing steps used for other surveys where such monetary valuations were not available:

- Quantity of own produce?
- Quantity of purchases?
- Quantity of gift received?
- Quantity of gift given?
- Expenditure on own-produced food in the last 30 days? (GH¢)
- Expenditure on purchased food in the last 30 days? (GH¢)
- Expenditure on gifts received in the last 30 days? (GH¢)
- Expenditure on gifts given in the last 30 days? (GH¢)
- Unit given?
- Number of months used?
- Total monthly expenditure on food? (GH¢)

### Data cleaning and variable construction

Processing each survey’s raw data involves several steps, including converting local measures into standard weight or volume units, determining unit prices, valuing consumption quantities, and constructing consistent socio-demographic variables.

### Local unit name harmonization

Survey respondents can report quantities in standard conventional units (kg, liter, etc.), as well as local non-standard units (mudu, bundles, bags, heaps, etc.), or both. Using conversion files collected by the survey teams, as much as possible, we convert the reported non-standard unit measures to standardized units. When conversion factors are unavailable and converting to conventional units is impossible, we harmonize non-standard units (e.g., we code Bundle, BUNDLE, bundled into bundle) to be able to capture all quantities in the unit price estimation. Missing conversion factors for some crops were imputed using medians for crops with available conversion factors at the lowest geographic level with at least 10 valid observations.

### Rationalize food item names

The description of food items consumed varies within country, reflecting local names and sub-crop varieties. For example, respondents in Ghana report consuming kenkey and banku, which are different forms of maize reported in their local names. In Kenya, variants of rice listed include aromatic and non-aromatic, white, brown, and broken rice. We rationalize food item names within the survey by aggregating the different forms of a specific food into a single category. For instance, in the case of Ghana, kenkey and banku, are aggregated into maize.

### Monetary valuation of food consumption in local currency units

Next, we estimate the monetary values of the reported quantities in local currencies. In all surveys, the value of purchased consumption is provided in each country’s domestic currency. However, in most surveys, the values of non-purchased consumption (own production and gifts) are not collected. To address this, we estimate item-specific prices, calculated as unit values of food purchases (value of purchases divided by the reported quantities of purchases converted into standard units). For items for which no purchase was reported, and for consumption from own production and gifts, we impute prices by estimating the median purchase price of the same food item-unit combination at the lowest administrative level with at least 10 observations. For example, when observations in the enumeration area (EA) are less than 10, we consider the median purchase price at the next administrative level (e.g., parish in Uganda, local government area in Nigeria, and ward in Tanzania) for which there are at least 10 observations. If there are still fewer than 10 valid unit prices at that level, this process continues until the median purchase price of a food item is determined at the country level. This iterative process allows for the full exploitation of available information to compute local prices for each household and food item-unit combination, rather than relying on a fixed price for every household. After estimating unit prices for each food item, we derive the value of food consumed from own production and gifts by multiplying reported quantities by the estimated unit price of each food item. A limitation of using unit prices to value food consumption instead of market prices is that they do not account for variations within food commodity groups or quality substitution bias, i.e., households or individuals that respond to price increases by switching to lower quality or cheaper produce^23^. Given the lack of market prices in the surveys, we rely on self-reported unit prices to value food consumption.

### Construct and merge selected socio-demographic variables

Each survey collects household demographic information in a household roster module. To improve the usability of the data, we construct household socio-economic and demographic variables. We include variables that capture the number of household members, female-headed households, the age of the household head, and the location of the household. We also generate dependency measures by constructing variables for the number of household members that are children (below 17 years), working age adults (>=18 and <65), female working age adults, male working age adults, and elders (>=65 years). To account for the crop season when data were collected, we construct variables for the day, month, and year the data collection interview was conducted. Survey weights provided in each survey wave are included to ensure the estimates are representative at the country level. Other socio-demographic variables included in the dataset are adult equivalence and households residing in rural areas. Finally, to facilitate spatial mapping, we include a variable that matches the first administrative unit where households reside to the corresponding administrative level, which is a commonly used country shapefile code from GADM. Although, this dataset does not include income variables, using codes in the EPAR repository^24^, income and other agricultural variables for all LSMS-ISA surveys for Ethiopia ESS, Nigeria GHS, Malawi IHS, Uganda NPS, and Tanzania NPS can be constructed and merged into the dataset using the household identifier variables.

### Data standardization across surveys

After the first set of steps to clean and construct consistent variables within each survey, the next stage involves steps to standardize across surveys such that the processed data have the same structure for all countries and survey waves.

### Aggregate original food items list into broad categories

The list of food items consumed varies substantially across countries, even across survey waves within a country. This reflects both the heterogeneity in local food consumption across countries and the choices made by the data collection agencies. We aggregate food items into broad categories to allow comparability across countries and waves. The food groups are: bananas and plantains, beef meat, cassava, dairy, eggs, fish and seafood, fruits, groundnuts, lamb and goat meat, maize, meals away from home, millet and sorghum, non-dairy beverages, nuts and seeds, oils and fats, other cereals, other meat, other roots and tubers, pork meat, potato, poultry meat, pulses, rice, spices, sugar, sweets, pastries, sweet potato, vegetables, wheat and yams. We also include a variable capturing a more compact grouping that has the categories: cereals, dairy, eggs, fish and seafood, fruits, vegetables, livestock products, spices, meals away from home, non-dairy beverages, oils and fats, processed foods, pulses, legumes, nuts, and roots and tubers. Table S3 in the Supplementary Information lists the components of each food category. The content of each category differs by country and survey. For example, in Ethiopia, oils and fats consist of butter, ghee, sunflower oil, and processed oils, whereas in Kenya, they include butter, ghee, margarine, groundnut butter, cooking fat, cooking oil, fortified cooking oil, lard, other oils, and fats.

### Annualize value of food consumption

Different surveys had different food consumption recall periods. For example, the LSMS-ISA surveys use a 7-day recall period while the Ghana Socio-economic Panel Survey uses a 30-day recall period. In order to standardize food consumption indicators across different survey countries and years, we annualize food consumption estimates. For surveys with a 7-day recall period, we multiply the food consumption value by 52 (the standard number of weeks in a year). For surveys with a 30-day recall period, we multiply the food consumption value by 12 (the number of months in a year). This could present limitations in the data because it ignores seasonal variations in food consumption, which is often very important in agricultural communities. A variable indicating the original recall period for each survey year is included in the dataset to allow users to account for this if necessary.

### Adjust monetary values for inflation and convert to 2017 PPP

To further facilitate comparisons across countries and surveys that take place in different years, we convert the nominal values calculated in local currencies into real values expressed in international dollars. Specifically, nominal values are first deflated using the National consumer price index (CPI) and then converted to 2017 Purchasing Power Parity (PPP) values. Both the CPI and PPP conversion factors are sourced from the World Bank’s World Development Indicators database.

### Address outliers through winzorisation

To mitigate extreme consumption values that could lead to biased and inefficient statistical estimates, we winsorize the consumption value by replacing the top 1% values with the 99th percentile.

### Append individual survey datasets into a combined dataset

Finally, all harmonized and standardized country-wave datasets are combined into a single, unified dataset, which includes all the food consumption indicators.

## Data Records

The harmonized dataset described in this article is available in a Figshare repository^25^: 10.6084/m9.figshare.29874011. It is deposited in two formats: a single Stata file and three csv files.

### Combined dataset of food consumption by items

The variables included in the combined dataset are listed in Table 2. We include several identification variables, including codes for administrative divisions, total food consumption value, food consumption value from purchase, food consumption value from own production, and food consumption value from gifts. The food consumption values are presented in nominal (local currency unit) and real (2017 PPP) values. We also include household demographic variables like rural location, age, gender of household head, number of household members, and the number of working and dependent household members.

**Table 2:** List of variables and their description.

Variable name	Variable Description
hhid	Household ID
weight	Household cross-section weight
GID_1	Adm1 code from the GADM shapefile
adm1	Administrative subdivision 1
adm2	Administrative subdivision 2
Instrument	Survey name
Year	Survey year
Country	Country name
interview_year	Household survey interview year
interview_month	Household survey interview month
interview_day	Household survey interview day
crop_category1	Aggregated Food Items 1
crop_category2	Aggregated Food Items 2
food_consu_value_ppp	Annual value of food consumed, 2017 PPP
food_consu_value_lcu	Annual value of food consumed, nominal LCU
food_purch_value_ppp	Annual value of food consumed from purchases, 2017 PPP
food_purch_value_lcu	Annual value of food consumed from purchased, nominal LCU
food_prod_value_ppp	Annual value of food consumed from own production, 2017 PPP
food_prod_value_lcu	Annual value of food consumed from own production, nominal LCU
food_gift_value_ppp	Annual value of food consumed from gifts, 2017 PPP
food_gift_value_lcu	Annual value of food consumed from gifts, nominal LCU
w_food_consu_value_ppp	Annual value of food consumed, 2017 PPP - Winzorized top 1%
w_food_consu_value_lcu	Annual value of food consumed, nominal LCU - Winzorized top 1%
w_food_purch_value_ppp	Annual value of food consumed from purchases, 2017 PPP - Winzorized top 1%
w_food_purch_value_lcu	Annual value of food consumed from purchases, nominal LCU - Winzorized top 1%
w_food_prod_value_ppp	Annual value of food consumed from own production, 2017 PPP - Winzorized top 1%
w_food_prod_value_lcu	Annual value of food consumed from own production, nominal LCU - Winzorized top 1%
w_food_gift_value_ppp	Annual value of food consumed from gifts, 2017 PPP - Winzorized top 1%
w_food_gift_value_lcu	Annual value of food consumed from gifts, nominal LCU - Winzorized top 1%
recall_period	Food consumption recall period in days
level_representativeness	Level of sub-national representativeness of the survey
conv_lcu_ppp	Conversion Factor
age_hh	Age of household head
fhh	Gender of household head
hh_members	Number of household members
adulteq	Adult-Equivalent
nadultworking	Number of working age adults in the household
nadultworking_female	Number of working age female adults in the household
nadultworking_male	Number of working age male adults in the household
nchildren	Number of children in the household
nelders	Number of elders in the household
Rural	Household resides in a rural area

## Data Overview

**Fig. 4:**
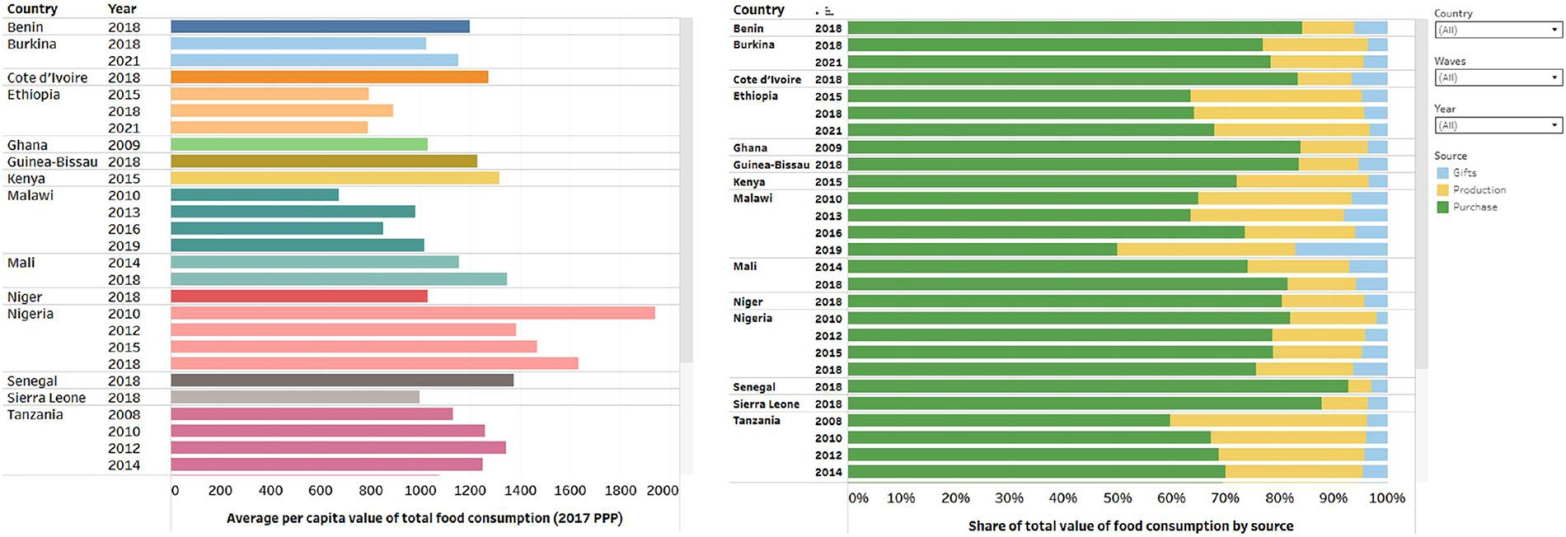
Mean total value and share of total value of per capita consumption from different sources.

This section illustrates some of the insights possible through the accompanying interactive Tableau visualization that rests on the harmonized dataset. Figure 4 presents graphs of the mean total per capita consumption and share of food consumption value from different sources. On average, Nigeria had the highest per capita value of food consumption in all years captured compared to the other SSA countries. About 75% of the household value of food consumed is from market purchases (in green), while own production (in yellow) and gifts (in blue) represent 20% and 5%, respectively. The data show substantial variations across countries and over time. Countries in East Africa show a greater tendency to source food from their own production than other regions of sub-Saharan Africa.

**Fig. 5:**
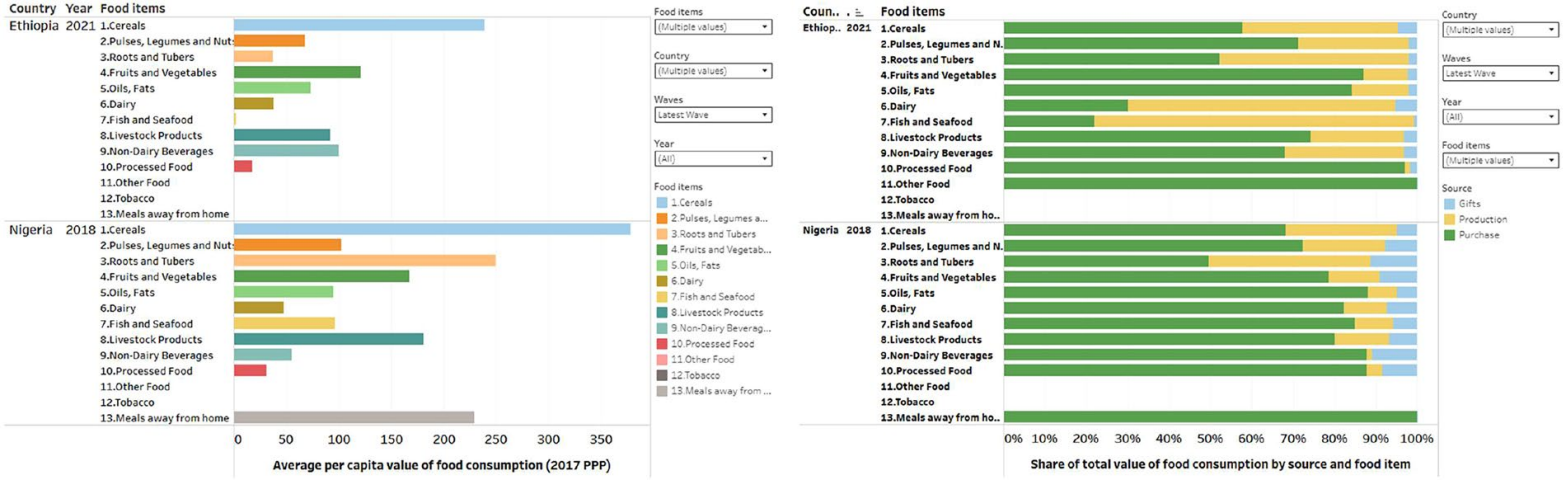
Mean value of consumption of different food categories and share of total value of consumption from different sources by food categories.

The dataset also allows disaggregation of consumption estimates by food category. In Fig. 5, we illustrate such a disaggregation for two surveys, Ethiopia 2021 and Nigeria 2018. The graph shows Cereals as the dominant food item, accounting for both countries’ highest per capita consumption value. The extent of reliance on market purchases for food acquisition varies by country and food commodity. For example, in Ethiopia, the majority of dairy consumption is from own production (in yellow, 64%), while in Nigeria, the majority is from purchases (in green, 82%). Further disaggregation of food consumption value and the share of food consumption value of various food categories by household location and the gender of the household head is possible.

## Technical Validation

To ensure the reliability and robustness of the harmonized food consumption dataset, we conducted multiple layers of technical validation to ensure the accuracy of data transformations, internal consistency, and statistical coherence across survey waves and countries.

### Source data integrity and reproducibility

All input datasets were obtained from publicly available, nationally representative household surveys such as the Living Standards Measurement Study – Integrated Surveys on Agriculture (LSMS-ISA). The LSMS-ISA and other surveys generally undergo rigorous methodological and technical validation before public release. During data collection, the World Bank and the country statistical offices implement a data quality assurance plan to validate data entries. The survey team also utilizes CAPI software, such as Survey Solutions, which flags data inconsistencies and potential errors during fieldwork and enables real-time, high-frequency data quality checks. All data transformations were implemented using reproducible Stata code, and the harmonization workflow is documented and version-controlled.

### Consistency and range checks

Internal consistency checks were applied to verify that key variables conformed to expected value ranges and logical relationships. We ensured that food values were expressed in consistent units across datasets, with local units converted to standard metric equivalents where necessary. The indicators were also carefully reviewed by inspecting summary statistics to detect any irregularities, and the code was thoroughly reviewed by at least two co-authors to ensure that any issues were not the result of coding errors.

### Outlier detection and treatment

We conducted an outlier analysis in which extreme values were visually examined using box plots and density plots. To mitigate the influence of outliers while preserving the underlying data structure, values in the top 1% of the distribution were winsorized. Summary statistics before and after winsorization were compared to assess the impact of the outlier-handling procedure.

### Cross-country and temporal validation

**Fig. 6:**
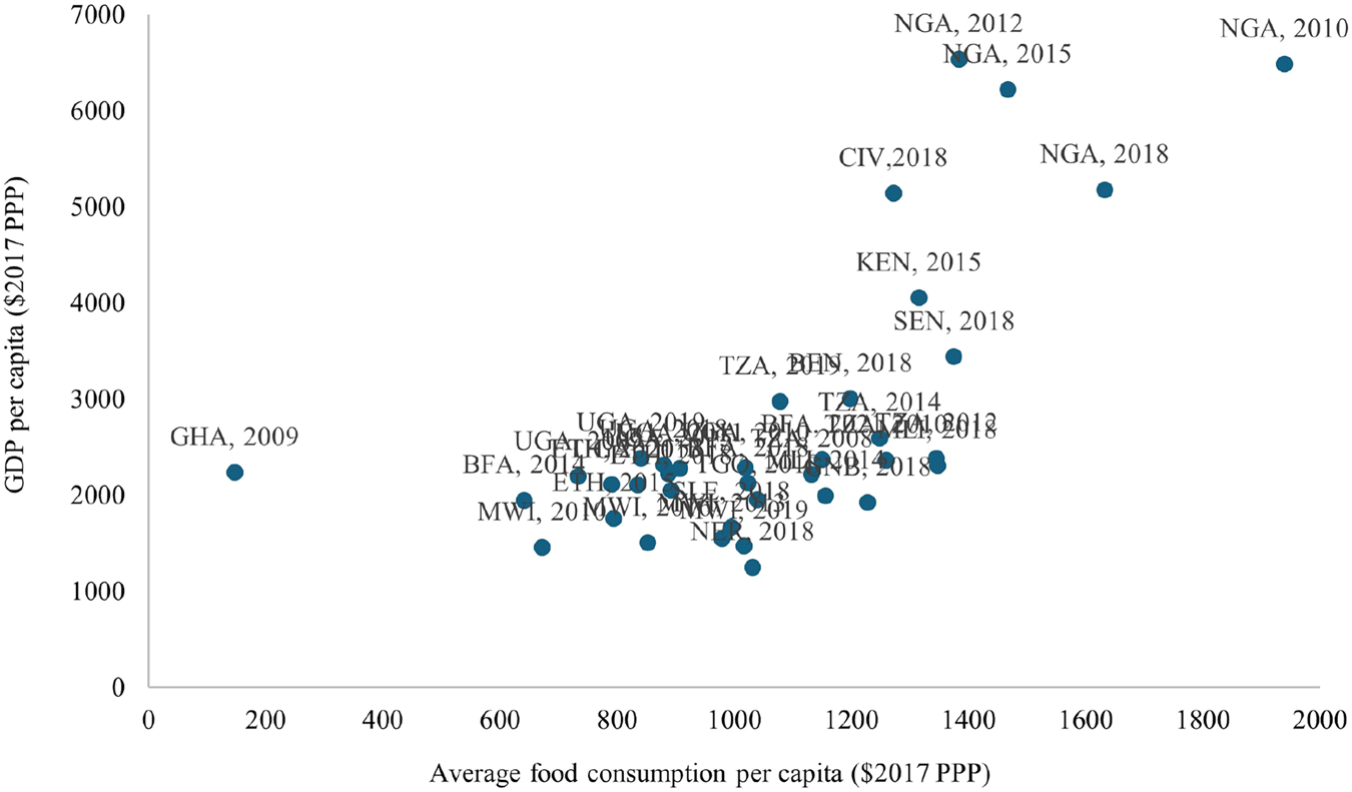
Graph showing convergence between the average food consumption value and GDP per capita for each country and year.

**Fig. 7:**
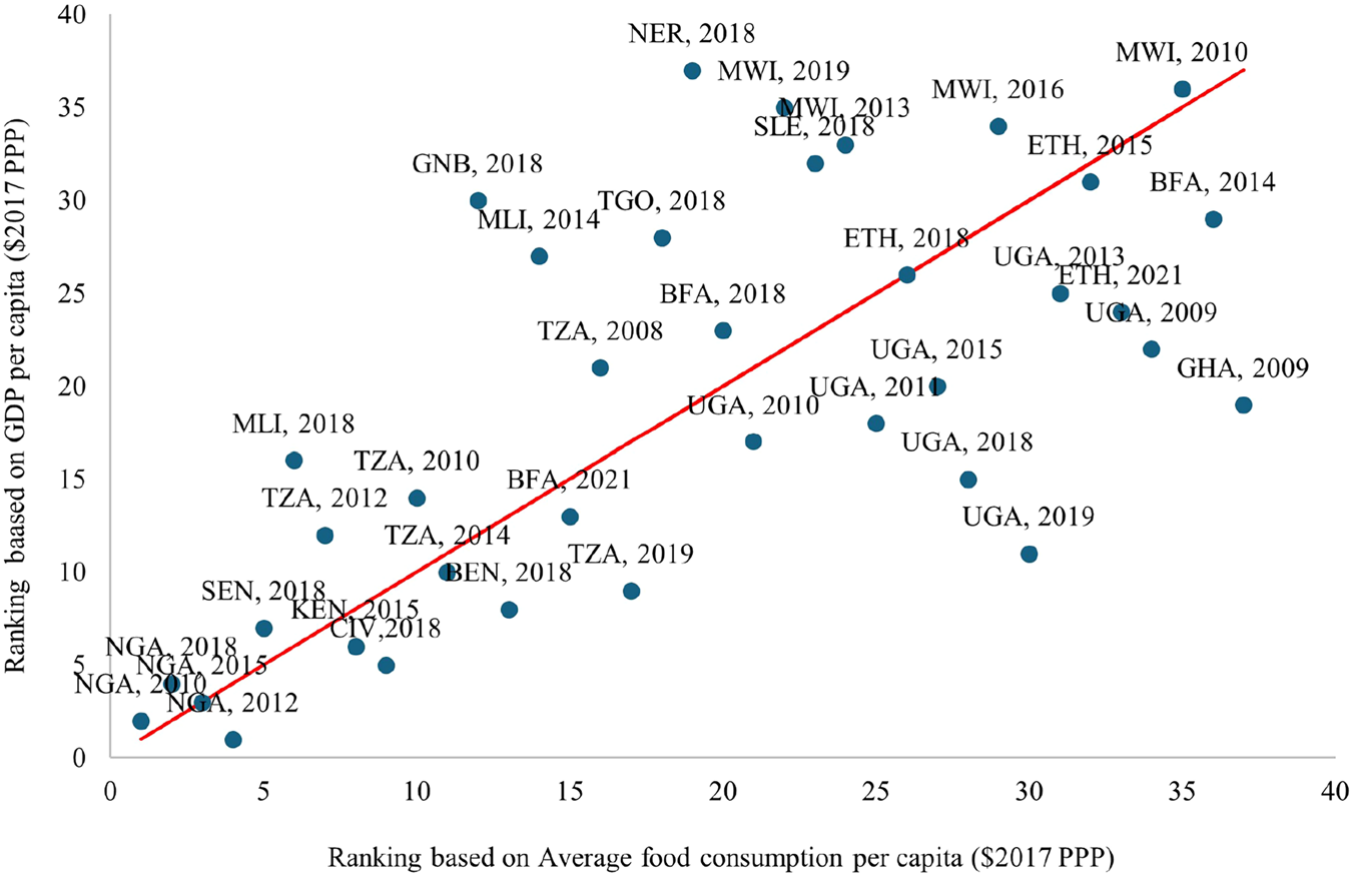
Graph showing convergence between the rankings of average food consumption value and GDP per capita for each country and year.

To validate the constructed food consumption indicators and further assess comparability across countries and survey years, we compare the average per capita value of food consumption to Gross Domestic Production per capita for matching survey years from the World Development Indicators database. Trends in consumption patterns over time were examined to ensure that observed changes were consistent with national economic and agricultural dynamics. Figure 6 shows a scatterplot of average per capita food consumption value and GDP per capita. As expected, we see a strong correlation between the two indicators. We also provide a scatterplot of countries’ ranking based on these two indicators (Fig. 7). These rankings are consistent, albeit with some differences, likely due to inherent noise in survey estimates and non-food consumption excluded from our dataset. Both scatterplots have a Pearson correlation coefficient of 0.4, statistically significant at the 5% level, indicating a tendency for higher average per capita food consumption value (consumption rankings) being associated with higher GDP per capita (GDP per capita rankings).

**Fig. 8:**
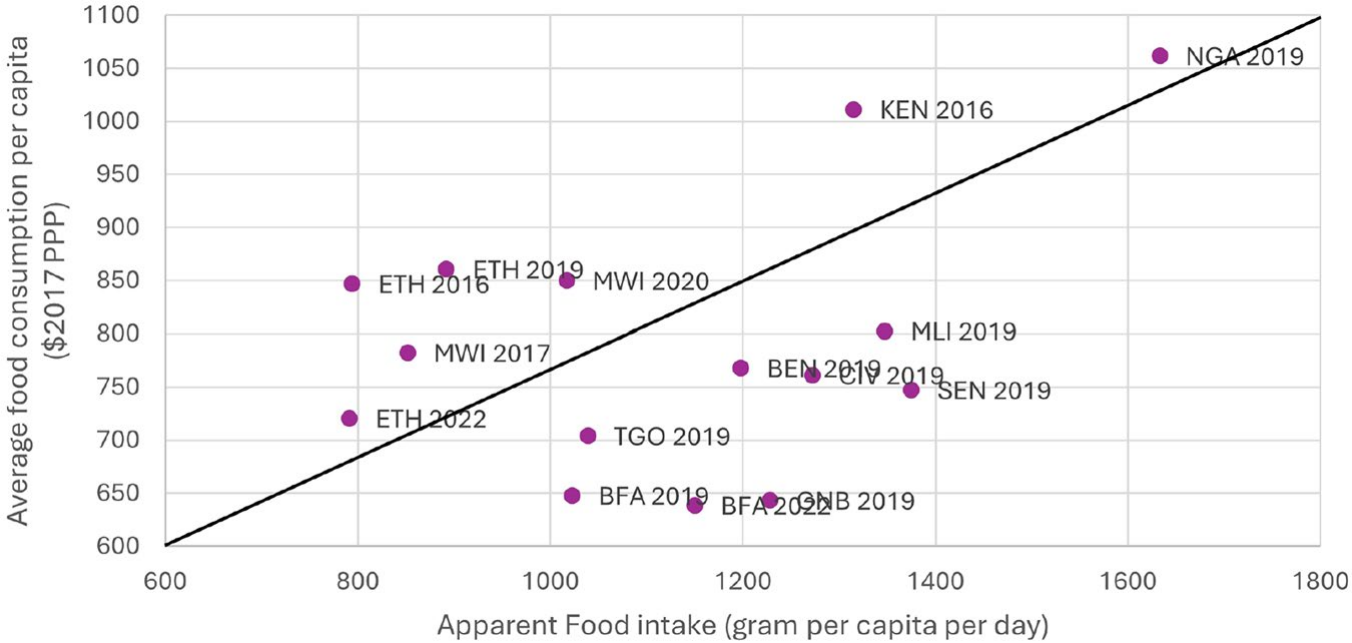
Graph showing convergence between the average food consumption value and aggregate FAOSTAT apparent food intake quantities for each country and year.

Our second validation exercise uses the FAOSTAT apparent food intake data, which reports the quantity of food consumption by food categories in grams per capita per day. The FAOSTAT data include food categories that are different from the categories captured in our consumption dataset. For this reason, we conduct the validation using aggregate consumption at the national level. Also, the apparent food intake data lacks price information to value quantities. Hence, they are not directly comparable to our estimates. Nonetheless, we plot a scatter plot of our estimated average value of food consumption, and FAOSTAT estimated aggregate per capita food intake quantities (Fig. 8). The scatter plot shows a moderate positive correlation between the two measures with a Pearson correlation coefficient of 0.36. This suggests that countries and years would be ranked similarly under either indicator.

We also rely on the Global Consumption Database (GCD), which includes two overlapping data points (Malawi 2010/11 and Uganda 2009/2010) for an additional validation. The GCD reported annual aggregate national value of household consumption. We convert these values into per capita consumption using population estimates from the World Development Indicators database. For Malawi, the average per capita value of food consumption in our dataset is $672 (2017 PPP), while the estimate based on GCD is $432 (2017 PPP). For Uganda, our estimate is $734 (2017 PPP), while the calculation using the GCD is $593 (2017 PPP). In both cases, our estimates are higher than the GCD estimates. These differences reflect variations in the data sources underlying the two estimates, as well as the use of aggregate national numbers in the GCD estimates as opposed to average per capita estimates in our case.

## Usage Notes

The household food consumption estimates constructed in this dataset are based on survey data from various secondary sources (See Table 1). Each survey wave is first processed separately, and identical key variables are generated for each wave, after which data for each wave is appended to construct the harmonized dataset. The harmonized dataset is at the crop level to facilitate consumption comparisons among major food categories. The data can be aggregated to the household level using the unique survey wave and household identifier variables (country_year and hhid) for household-level analysis. Variables identifying the first and second administrative level of household residence were also included in the dataset (*adm1* and *adm2*). To obtain nationally representative estimates and account for complex survey data design, users must use sample weights (*weight*) as probability weights and strata identifiers to obtain valid standard errors.

## Supplementary information

Supplementary information

## Data Availability

The entire dataset is available online in a Figshare repository. The following URL provides access to the dataset^25^: 10.6084/m9.figshare.29874011.

## Code availability

The Stata codes used to process the raw data, generate standardized food consumption indicators, and append different survey waves for each country into a combined dataset are available on GitHub (https://github.com/EvansSchoolPolicyAnalysisAndResearch/Household-Consumption-Data). Each survey data processed has its own Stata dofile. Additionally, we include a dofile used to append individual datasets into a combined dataset. The do files are annotated to describe and guide the user through the different stages of data processing. This ensures full reproducibility of the data harmonization process and enables users to trace each indicator back to its original source. An interactive and more comprehensive visualization of the data is accessible on the Tableau visualization platform. Table S4 in the Supplementary Information lists the names of Stata code files available in the GitHub repository.
