# Supplementary material for: Harmonized food consumption dataset by food category and acquisition source for Sub-Saharan African countries
Source: Sci Data. 2026 Jan 21;13:232. doi: 10.1038/s41597-026-06548-1 (PMC12902005; doi:10.1038/s41597-026-06548-1)
Supplement: Supplementary file 1 — Supplementary information [file 41597_2026_6548_MOESM1_ESM.pdf]

Supplementary Information to Harmonized food consumption dataset by food category and acquisition source for Sub-Saharan African countries

Table of Contents

Table S1: Inclusion and exclusion criteria for countries included in the harmonized dataset…………………………….2

Table S2: Links to raw data ……………………………………………………………………………..…………..3

Table S3: Food item categorization for food_category2……………………………………………………..…….....4

Table S4: List of Stata code files available in GitHub repository………………………………………...……….5

Table S1: Inclusion and exclusion criteria for countries included in the harmonized dataset

Countries	Inclusion	Reason for inclusion/exclusion
Angola	No	No publicly available household consumption survey online
Benin	Yes	Publicly available
Botswana	No	No publicly available household consumption survey online
Burkina Faso	Yes	Publicly available
Burundi	No	No publicly available household consumption survey online
Cameroon	No	No publicly available household consumption survey online
Cape Verde	No	No publicly available household consumption survey online
Central African Republic	No	No publicly available household consumption survey online
Chad	No	No publicly available household consumption survey online
Comoros	No	No publicly available household consumption survey online
Dem. Rep. of the Congo	No	No publicly available household consumption survey online
Djibouti	No	No publicly available household consumption survey online
Equatorial Guinea	No	No publicly available household consumption survey online
Eritrea	No	No publicly available household consumption survey online
Ethiopia	Yes	Publicly available
Gabon	No	No publicly available household consumption survey online
Gambia	No	Consumption data files were multiple and different
Ghana	Yes	Publicly available
Guinea	No	No publicly available household consumption survey online
Guinea-Bissau	Yes	Publicly available
Côte d'Ivoire	Yes	Publicly available
Kenya	Yes	Publicly available
Lesotho	No	No publicly available household consumption survey online
Liberia	No	Publicly available, but consumption values are too high
Madagascar	No	No publicly available household consumption survey online
Malawi	Yes	Publicly available
Mali	Yes	Publicly available
Mauritania	No	No publicly available household consumption survey online
Mauritius	No	No publicly available household consumption survey online
Mayotte	No	No publicly available household consumption survey online
Mozambique	No	No publicly available household consumption survey online
Namibia	No	No publicly available household consumption survey online
Niger	Yes	Publicly available
Nigeria	Yes	Publicly available
Republic of the Congo	No	No publicly available household consumption survey online
Rwanda	No	Consumption data is not disaggregated by source
São Tomé and Principe	No	No publicly available household consumption survey online
Senegal	Yes	Publicly available
Seychelles	No	No publicly available household consumption survey online
Sierra Leone	Yes	Publicly available
Somalia	No	No publicly available household consumption survey online
South Africa	No	Consumption data is not disaggregated by source
South Sudan	No	No publicly available household consumption survey online
Swaziland	No	No publicly available household consumption survey online
Tanzania	Yes	Publicly available
Togo	Yes	Publicly available
Uganda	Yes	Publicly available
Zambia	No	No publicly available household consumption survey online
Zimbabwe	No	No publicly available household consumption survey online

Table S2: Links to raw data

Country	Survey wave	Links
Benin	2018/19 Enquête Harmonisée sur les Conditions de Vie des Ménages (EHCVM)	https://microdata.worldbank.org/index.php/catalog/4291
Burkina Faso	2018/19 EHCVM	https://microdata.worldbank.org/index.php/catalog/4290
	2021/21 EHCVM	https://microdata.worldbank.org/index.php/catalog/6224
Cote d'Ivoire	2018/19 EHCVM	https://microdata.worldbank.org/index.php/catalog/4292
Ethiopia	2015/16 Socioeconomic Panel Survey (ESS)	https://microdata.worldbank.org/index.php/catalog/2783
	2018/19 ESS	https://microdata.worldbank.org/index.php/catalog/3823
	2012/22 ESS	https://microdata.worldbank.org/index.php/catalog/6161
Ghana	2009/10 Socioeconomic Panel Survey (SPS)	https://microdata.worldbank.org/index.php/catalog/2534
Guinea Bissau	2018/19 EHCVM	https://microdata.worldbank.org/index.php/catalog/4293
Kenya	2015/16 Integrated Household Budget Survey (KIHBS)	https://statistics.knbs.or.ke/nada/index.php/catalog/13
Malawi	2010/11 Integrated Household Survey (IHS)	https://microdata.worldbank.org/index.php/catalog/1003
	2013 IHS	https://microdata.worldbank.org/index.php/catalog/2248
	2016/17 IHS	https://microdata.worldbank.org/index.php/catalog/2936
	2019/20 IHS	https://microdata.worldbank.org/index.php/catalog/3818
Mali	2014 Enquête Agricole de Conjoncture Intégrée (EACI)	https://microdata.worldbank.org/index.php/catalog/2583
	2018/19 EHCVM	https://microdata.worldbank.org/index.php/catalog/4295
Niger	2018/19 EHCVM	https://microdata.worldbank.org/index.php/catalog/4296
Nigeria	2010/11 General Household Survey (GHS)	https://microdata.worldbank.org/index.php/catalog/1002
	2012/13 GHS	https://microdata.worldbank.org/index.php/catalog/1952
	2015/16 GHS	https://microdata.worldbank.org/index.php/catalog/2734
	2018/19 GHS	https://microdata.worldbank.org/index.php/catalog/3557
Senegal	2018/19 EHCVM	https://microdata.worldbank.org/index.php/catalog/4297
Sierra Leone	2018 Integrated Household Survey (IHS)	https://catalog.ihsn.org/catalog/9246
Tanzania	2008/09 National Panel Survey (NPS)	https://microdata.worldbank.org/index.php/catalog/76
	2010/11 NPS	https://microdata.worldbank.org/index.php/catalog/1050
	2012/13 NPS	https://microdata.worldbank.org/index.php/catalog/2252
	2014/15 NPS	https://microdata.worldbank.org/index.php/catalog/2862
	2019/20 NPS	https://microdata.worldbank.org/index.php/catalog/3885
Togo	2018/19 EHCVM	https://microdata.worldbank.org/index.php/catalog/4298
Uganda	2009/10 National Panel Survey (UNPS)	https://microdata.worldbank.org/index.php/catalog/1001
	2010/11UNPS	https://microdata.worldbank.org/index.php/catalog/2166
	2011/12 UNPS	https://microdata.worldbank.org/index.php/catalog/2059
	2013/14 UNPS	https://microdata.worldbank.org/index.php/catalog/2663
	2015/16 UNPS	https://microdata.worldbank.org/index.php/catalog/3460
	2018/19 UNPS	https://microdata.worldbank.org/index.php/catalog/3795
	2019/20 UNPS	https://microdata.worldbank.org/index.php/catalog/3902

Table S3: Food item categorization for food_category2

	Main crop category	Food items
1	Cereals	Rice (aromatic, non-aromatic, white, brown, broken, local, imported), maize (green, grain, flour, popcorn, horse corn, fortified, flour, dough, kenkey, banku), guinea corn, millet (grain, flour), fonio, sorghum (grain, flour), wheat (grain, white, brown, fortified, spaghetti, macaroni, pasta), teff, injera, oats, barley, breakfast cereal, baby food cereals, and other cereals.
2	Dairy	Fresh milk, curdled milk, buttermilk, goat milk, camel milk, condensed milk, powdered milk, cheese, baby milk, sour milk, yoghurt, other dairy products, and eggs.
3	Fish and seafood	Different types of fish (fresh, canned, frozen, dried, smoked), crabs, shrimps, prawns, lobster, and other seafood.
4	Fruits and vegetables	Mango, pineapple, orange, paw paw, ripe banana, apple, lemons, avocados, watermelon, passion fruit, pears, peaches, plums, grapefruit, grapes, strawberries, melons, dates, coconut, sugar cane, guavas, lime, loquats, tangerines, tree tomato, other fruits and berries, leeks, onions, cabbage, chinese cabbage, carrot, tomatoes, cucumber, french beans, green bean, runner beans, peas, lettuce, celery, beetroots, courgettes, squash, marrow, capsicum, pepper (fresh, dried), okra (fresh, dried), onion, leaves (sorrel, gourd, bean, cassava, taro, baobab, potato, cocoyam, spinach, kale, other), broccoli, radish, mushroom, eggplant, zucchini, garden eggs, pumpkins, coriander leaves, turnips, tinned vegetables, other vegetables, spices (garlic, ginger, salt, mayonnaise, vinegar/mustard, yeast, chili sauce, other condiments).
5	Livestock products	Meats (Beef, camel, mutton, goat, offal and tripe, pork, chicken, guinea fowl, duck, rabbit, mice, ham, salami, corned beef, sausages, bacon, other domestic poultry), wild game, termites, other insects, and other meats.
6	Meals away from home	Meals away from home.
7	Non-dairy beverages	Coffee, tea, milo, cocoa and cocoa products, soy drinks, fruit juice, herbal tea and infusions, mineral water, filtered water, bottled water, soft drinks, squashes, sodas, energy drinks, vegetable juice, juice powder, sobo drink, other drinks, vodka, rum, whisky, brandy, palm wine, pito, gin, local liquor, other spirits, wine, sparkling wines, fortified wines, beers, traditional beers, industrial beers, and other alcoholic beverages.
8	Oils and fats	Butter, shea butter, ghee, margarine, groundnut oil, coconut oil, palm oil, cottonseed oil, palm kernel oil, cooking fat, cooking oil, fortified cooking oil, processed oils, lard, soybean oil, vegetable oil, other oils and fats.
9	Processed foods	Bread (white, brown), croissants, cookies, cakes, biscuits, jaggery, jam, jelly, syrups, marmalade, honey, doughnuts, pancakes, chocolate bar, chocolate spread, sugar, icing sugar, caramel, sweets, chewing gum, and other sugar confectioneries.
10	Pulses, legumes, nuts	Groundnuts (fresh, shelled, dried, roasted, paste), sesame, almonds, cashew nuts, shea nut, soya beans (seed, flour), horsebeans, field pea, chick pea, black eyed beans, lentils, haricot beans, other pulses, fenugreek, vetch, niger seed, linseed, beans (white, brown), pigeon peas, green grams, dolicos, other grams, peas, macadamia nuts, cowpeas, chickpeas, tinned beans, kolanut, and other pulses, nuts, and legumes.
11	Roots and tubers	Cassava (dried, fresh, flour, tapioca, garri, root), yam, cocoyam, potato, sweet potato(white, yellow, fresh, dried, flour), arrow root, taro, cooking bananas, plantain, other roots and tubers.
12	Tobacco	Cigarette, cigar, tobacco (pipe, raw, snuff, chewing, smoking), miraa, cannabis, bangi-cannabis, glue, other stimulants, and narcotics.
13	Other food	Breakfast, lunch, and dinner taken outside, and other meals.

Table S4: List of Stata code files available in GitHub repository

Country	Survey	Stata code name
Benin	Enquête Harmonisée sur les Conditions de Vie des Ménages (EHCVM) 2018/19	BeninW1_Food Consumption by source.do
Burkina Faso	2018/19 EHCVM	BurkinaW2_Food Consumption by source.do
	2021/22 EHCVM	BurkinaW3_Food Consumption by source.do
Cote d'Ivoire	2018/19 EHCVM	Cote d'IvoireW1_Food Consumption by source.do
Ethiopia	2015/16 Ethiopia Socioeconomic Panel Survey (ESS)	EthiopiaW3_Food Consumption by source.do
	2018/19 ESS	EthiopiaW4_Food Consumption by source.do
	2012/22 ESS	EthiopiaW5_Food Consumption by source.do
Ghana	2009/10 Socioeconomic Panel Survey (SPS)	GhanaW1_Food Consumption by source.do
Guinea Bissau	2018/19 EHCVM	Guinea-BissauW1_Food Consumption by source.do
Kenya	2015/16 Integrated Household Budget Survey (KIHBS)	KenyaW1_Food Consumption by source.do
Malawi	2010/11 Integrated Household Survey (IHS)	Malawi_W1_Food Consumption by source.do
	2013 IHS	Malawi_W2_Food Consumption by source.do
	2016/17 IHS	Malawi_W3_Food Consumption by source.do
	2019/2020 IHS	Malawi_W4_Food Consumption by source.do
Mali	2014 Enquête Agricole de Conjoncture Intégrée (EACI)	Mali_W1_Food Consumption by source.do
	2018/19 EHCVM	Mali_W2_Food Consumption by source.do
Niger	2018/19 EHCVM	Niger_W3_Food Consumption by source.do
Nigeria	2010/22 General Household Survey (GHS)	Nigeria_W1_Food Consumption by source.do
	2012/13 GHS	Nigeria_W2_Food Consumption by source.do
	2015/16 GHS	Nigeria_W3_Food Consumption by source.do
	2018/19 GHS	Nigeria_W4_Food Consumption by source.do
Senegal	2018/19 EHCVM	Senegal_W1_Food Consumption by source.do
Sierra Leone	2018 Integrated Household Survey (IHS)	Sierra Leone_W3_Food Consumption by source.do
Tanzania	2008/09 National Panel Survey (NPS)	Tanzania_W1_Food Consumption by source.do
	2010/11 NPS	Tanzania_W2_Food Consumption by source.do
	2012/13 NPS	Tanzania_W3_Food Consumption by source.do

	2014/15 NPS	Tanzania_W4_Food Consumption by source.do
	2019/20 NPS	Tanzania_W5_Food Consumption by source.do
Togo	2018/19 EHCVM	Togo_W1_Food Consumption by source.do
Uganda	2009/10 National Panel Survey (UNPS)	Uganda_W1_Food Consumption by source.do
	2010/11 UNPS	Uganda_W2_Food Consumption by source.do
	2011/12 UNPS	Uganda_W3_Food Consumption by source.do
	2013/14 UNPS	Uganda_W4_Food Consumption by source.do
	2015/16 UNPS	Uganda_W5_Food Consumption by source.do
	2018/19 UNPS	Uganda_W7_Food Consumption by source.do
	2019/20 UNPS	Uganda_W8_Food Consumption by source.do
Combined dataset		Append all surveys_SSA.do
